# Weighting of Spatial and Spectro-Temporal Cues for Auditory Scene Analysis by Human Listeners

**DOI:** 10.1371/journal.pone.0059815

**Published:** 2013-03-19

**Authors:** Peter Bremen, John C. Middlebrooks

**Affiliations:** 1 Department of Otolaryngology, University of California Irvine, Irvine, California, United States of America; 2 Department of Neurobiology and Behavior, University of California Irvine, Irvine, California, United States of America; 3 Department of Cognitive Sciences, University of California Irvine, Irvine, California, United States of America; 4 Department of Biomedical Engineering, University of California Irvine, Irvine, California, United States of America; 5 Center for Hearing Research, University of California Irvine, Irvine, California, United States of America; University of Salamanca- Institute for Neuroscience of Castille and Leon and Medical School, Spain

## Abstract

The auditory system creates a neuronal representation of the acoustic world based on spectral and temporal cues present at the listener's ears, including cues that potentially signal the locations of sounds. Discrimination of concurrent sounds from multiple sources is especially challenging. The current study is part of an effort to better understand the neuronal mechanisms governing this process, which has been termed “auditory scene analysis”. In particular, we are interested in spatial release from masking by which spatial cues can segregate signals from other competing sounds, thereby overcoming the tendency of overlapping spectra and/or common temporal envelopes to fuse signals with maskers. We studied detection of pulsed tones in free-field conditions in the presence of concurrent multi-tone non-speech maskers. In “energetic” masking conditions, in which the frequencies of maskers fell within the ±1/3-octave band containing the signal, spatial release from masking at low frequencies (∼600 Hz) was found to be about 10 dB. In contrast, negligible spatial release from energetic masking was seen at high frequencies (∼4000 Hz). We observed robust spatial release from masking in broadband “informational” masking conditions, in which listeners could confuse signal with masker even though there was no spectral overlap. Substantial spatial release was observed in conditions in which the onsets of the signal and all masker components were synchronized, and spatial release was even greater under asynchronous conditions. Spatial cues limited to high frequencies (>1500 Hz), which could have included interaural level differences and the better-ear effect, produced only limited improvement in signal detection. Substantially greater improvement was seen for low-frequency sounds, for which interaural time differences are the dominant spatial cue.

## Introduction

The everyday acoustic environment is complex, in that multiple independent sound sources may be active at any given moment in time. Since the number of individual sources is *a priori* unknown, segregation of sources in a mixture is a computationally ill-posed problem with infinite solutions. To solve this problem, the auditory system must employ heuristics in order to constrain the space of possible solutions. This process has been termed auditory scene analysis (ASA) [1] and is thought to be based on properties present in naturally occurring sounds (e.g. vocalizations) such as common onset/offset, common modulation, harmonicity, and common location of sound elements. When two acoustic sources share some of these properties (cues) they tend to be grouped together and to be perceived as one auditory object (perceptual fusion). When, on the other hand, sources differ sufficiently from each other along the cue dimensions they will be segregated and consequently will be perceived as distinct auditory objects (perceptual fission).

To gain a better understanding of each cue's relative contribution to perception we studied the detection of signals in the presence of various interfering (i.e., masking) sounds with an emphasis on spatial cues. Our assumption is that detection of the signal is enhanced when the signal and masker are perceived as different auditory objects. Intuitively, one might think that sound source location would contribute strongly to perceptual fission, as for example, spatial separation of a talker and background babble facilitates conversation at a crowded cocktail party [2], [3]. Sound-source location itself, however, is not mapped at the auditory periphery and, instead, must be computed from multiple binaural and monaural cues arising from the interaction of sound with the head and external ears [4]. For example, binaural difference cues, i.e. interaural time differences (ITDs) for low-frequency (approx. <1.5 kHz) and interaural level differences (ILDs) for high-frequency (approx. >3 kHz) sound localization in azimuth, need to be extracted in specialized pathways along the neuraxis [5], [6]. This calculation can be prone to error, and spatial cues can be degraded by reverberation [7], [8]. One might conjecture, therefore, that the auditory system would put less weight on binaural cues for ASA than on spectro-temporal cues, which are directly encoded along the basilar membrane of the cochlea and in the timing of action potentials. Indeed, results reported in the literature are ambiguous regarding the importance of spatial cues for ASA, with some authors arguing that they play only a minor role under some conditions [9], [10], [11] whereas other investigator demonstrated clear spatial effects [12], [13], [14].

We tested the contributions of temporal, spectral and spatial cues to signal detection in the presence of various perceptually distinct non-speech maskers. Experiments were conducted in free-field, anechoic conditions. Spatial separation of signal and masker had relatively little influence on signal detection in “energetic” masking conditions in which the signal frequency fell within the band containing the masker frequencies. In contrast, spatial separation of signal and masker resulted in substantial improvement in signal detection (i.e., “spatial release from masking”) in “informational masking” conditions in which signal and maskers were separated in frequency but in which listeners might have confused signal with masker. Spatial release from masking was markedly greater for low-frequency sounds (<1.5 kHz), in which ITDs are the dominant cue, than for high frequencies, in which ILDs and the better-ear effect might have aided detection. Low-frequency spatial cues could even overcome the tendency of common onsets to fuse signal with masker.

## Materials and Methods

### Ethics Statement

The experiments were undertaken with the understanding and written consent of each subject, following the Code of Ethics of the World Medical Association (Declaration of Helsinki) and were approved by the Institutional Review Board of the University of California Irvine (UCI).

### Listeners

Thirteen paid volunteers (19–27 years; mean 22 years; 10 females; right hand dominant except for S57) recruited from the UCI student body participated in the study. Listeners' thresholds were within 20 dB of audiometric zero for frequencies between 0.25 and 8 kHz. Two of the listeners (S36 and S42) participated in all of the experiments in this study, three listeners (S32, S33 and S55) participated in three of the four experiments, and all others participated in two experiments. To facilitate comparison, we ordered subjects according to their ID number (S31, S32, S33, S36, S37, S39, S42, S54, S55, S56, S57, S58, S59) in all figures.

### Stimuli

All stimuli were generated digitally at 50 kHz with 24-bit precision using System 3 equipment from Tucker-Davis Technologies. The 3.5-in 2-way coaxial speakers were calibrated with tones from 0.2 to 14.4 kHz in 1/6-oct steps with a precision microphone positioned at the usual location of the listener's head, and a correction table was stored for each speaker. All signal and masker components consisted of multiple tone bursts shaped with a 50-ms Gaussian envelope (σ = 6.25 ms). We choose a Gaussian envelope rather than a cosine ramp in order to minimize spectral splatter. Note that the Gaussian envelope concentrated most of the sound energy in a narrow window of about 10 ms. Each sound presentation consisted of 4 complex bursts at a rate of 10/s. Each complex burst comprised four masker frequencies plus, on half of the presentations, a single signal frequency. The signal, when present, consisted of four bursts at a constant frequency (i.e., one signal burst as part of each of the 4 complex bursts). The frequency composition of the masker bursts was constant or varied among bursts, as described below. Maskers were presented in synchronous and asynchronous conditions. In the synchronous condition, all masker components were gated on simultaneously with the signal. In the asynchronous condition, 4 delays were assigned randomly to the 4 masker components. Delay values were 0, 25, 50, 75 ms relative to stimulus onset, which was defined as 0 ms. In asynchronous conditions, therefore, one masker component overlapped with the signal and the other three were largely separated in time from the signal and from each other. All maskers were created fresh for each stimulus, i.e. the listeners never heard the same masker twice.

Signal and masker differed in spectral composition. The signal frequency was either 600 Hz or 4000 Hz. The masker complex comprised four tones, each tone presented at 45 dB SPL, and masker frequencies could be distributed across the spectrum in a number of ways. We divided the frequency spectrum in bands spaced equally on a logarithmic scale (see below). Masker frequencies were then drawn randomly from these bands in order to create two classes of maskers, namely energetic and informational maskers [15]. In the case of energetic masking, signal and masker energy overlapped in the same auditory filters and, presumably, no processing strategy could disentangle them. We constructed energetic maskers whose components fell within a band of 1/3 octaves below and above the signal frequency; energetic maskers are denoted here with a prefix “e”. In the case of informational masking, signal and masker components lay in different peripheral channels, and masking was thought to result from computational processes related to ASA in more central stages of the auditory system [16]. We constructed informational maskers in which masker components were excluded from a protected band extending 1/3 oct above and below the signal frequency; informational maskers are denoted with a prefix “i”. Both energetic and informational maskers were generated in two types. For the first type, components were drawn independently, i.e. randomly, for each of the signal pulses. This stimulus has been termed multiple-burst different in the informational masking literature, and we denote it here as “R” [17]. For the second type, four masker components were drawn for each trial, and those four components remained constant for all of the stimulus bursts [17]. This masker type has been termed multiple-burst same in the literature, and we denote it here as “C”. The R and C maskers were perceptually quite different, with the R type resembling a bird's twitter and the C type resembling a repeated complex tone.

We tested various spatial configurations of signal and masker. These spatial configurations are represented with the nomenclature SxMx, with S and M indicating the locations of the signal and masker, respectively. The x indicates azimuth location in degrees to the right of the subject and could assume the following values 0, 10, 15, 20, 40, 80, with “no” meaning not present. The signal was always presented at 0°, directly in front of the listener. We used the S0Mno condition to measure the detection thresholds in quiet, T_Q_, for both signal frequencies used. The iR, eR and eC masker backgrounds were tested only in S0M0 and S0M80 configurations, whereas we tested all S0Mx spatial configurations for the iC masker.

In order to isolate the contributions of ITDs or ILDs, we created informational maskers spanning three ranges of frequencies. In the all-pass condition, masker components were drawn from 38 masker bands ranging from 0.2 to 14 kHz in steps of 1/6 of an octave; those were the same frequencies used for calibration. The all-pass masker potentially contained ITD, ILD and better-ear (head-shadow effect) spatial cues. In the low-pass condition, masker components were drawn from a total of 10 frequencies ranging from 0.2 to 1.5 kHz in steps of 1/6 oct outside of the protected band of 449 to 713 Hz. In this case the predominant spatial cue presumably was ITD. In the high-pass condition, masker components were drawn from 13 frequencies ranging from 1.5 to 12 kHz in steps of 1/6 oct not including frequencies within the protected band of 3.2 to 5.1 kHz. In the high-pass condition, listeners might have utilized ILDs, the head-shadow effect, or ITDs in sound envelopes. Note that none of the frequencies in the band around 1.5 to 3 kHz carry strong spatial cues because ILDs tend to be small, less than a few decibels, and because listeners are insensitive to interaural delays in temporal fine structure. That (1.5 to 3 kHz) band was included in the high-pass masker for the purpose of equalizing its octave frequency range with that of the low-pass masker. We tested the all-pass masker with both the 600-Hz and 4000-Hz signal, the low-pass masker only with the 600-Hz signal and the high-pass masker only with the 4000-Hz signal. The energetic maskers were necessarily low-pass or high-pass because all the components clustered around the 600- or 4000-Hz signal.

### Procedure

Experiments were conducted in a double-walled sound attenuating chamber (IAC, inner dimensions: 2.6 m ×2.6 m ×2.7 m) lined with SONEXone absorbent foam. The chamber contained a circular hoop, 1.25 m in radius, positioned in the horizontal plane at the height of the listener's inter-aural axis. The listener sat in the center of the hoop on a rotatable straight-back chair and were instructed to keep their head and eyes facing straight ahead during stimulus presentation. Although we did not routinely monitor head positions, given the brief stimulus duration we think it unlikely that the listeners were able to use dynamic head movement cues to solve the task. All sessions were performed in darkness to exclude other sensory cues.

We used a two-interval, two-alternative, forced-choice (2AFC) test with a 3-down 1-up scheme to measure the free-field signal detection threshold of the listeners. This procedure tracks the 79% correct point on the psychometric curve [18]. Signal level was adjusted by 3 dB SPL for the first three reversals and in steps of 1 dB SPL thereafter. An experimental run ended after 10 reversals and the threshold was determined as the average of the last 6 reversals. We repeated the threshold measurements at least 8 times per listener (except for S31 in the all-pass 600 Hz condition: 4 repetitions). Listeners indicated the interval in which they detected the signal by pressing one of two buttons on a hand-held response box (TDT). Light-emitting diodes (LEDs) on the response box were used to indicate the beginning of a trial, the current interval and, during training (see below), to provide feedback to the listener.

Before starting the experiments we trained the listeners in the following manner. We initially presented the signal without a masker, i.e. one interval contained the signal and the other interval contained no sound at all. After each trial we provided feedback to the listener by flashing the LED that corresponded to the signal interval. That run served to familiarize the subject with the signal. Next we tested with a masker at a level of 25 dB SPL and a spatial configuration of S0M80. After obtaining a threshold value for this masker level we repeated training at increased masker levels (35 and 45 dB SPL). Typically, thresholds for the iC maskers would increase systematically with increasing masker level, whereas they would stay relatively constant for the iR maskers, indicating a floor effect. After reaching the masker level to be used in the experiments (45 dB SPL), we changed the spatial configuration to S0M0 and repeated the training on both the iR and the iC maskers.

Data for each condition were collected over multiple sessions stretched over several months. In a session of 90 minutes listeners would on average complete about 10 blocks (∼100 trials) with short breaks in between blocks. Within a block lasting about 5 minutes we kept signal frequency (either 600 Hz or 4000 Hz), signal location (always 0°), masker type (either iR or iC or eR or eC) and masker location (either 0° or 10° or 15° or 20° or 40° or 80°) constant. Masker type and masker location were randomized between blocks. We started with all-pass maskers and a 4000-Hz signal (S32, S36, S37, S39, S42, S57, S58). Next, in one half of the subjects (S31, S39, S42, S55) we first tested the low-pass maskers with the 600-Hz signal before testing the high-pass maskers with the 4000-Hz signal. In the other half of the subjects (S32, S33, S36, S54, S56, S59) we switched the order. Finally, we tested all-pass maskers with a 600-Hz signal (S31, S33, S36, S42, S55, S57, S58, S59). We recruited subjects S57, S58 and S59 towards the end of the study. Therefore, S57 and S58 were tested with the all-pass maskers with the 4000-Hz signal and S59 was tested with the high-pass maskers after their data for the all-pass masker with the 600-Hz signal had been collected.

### Data Analysis

We referenced all detection thresholds to T_Q_, i.e., the threshold in quiet, by subtracting T_Q_ from the threshold in the presence of the masker. As a result T_Q_ is written as 0 dB for both signal frequencies. Thresholds for any particular masker condition tended not to be normally distributed across listeners. For that reason, we used non-parametric statistical tests to compare distributions of thresholds among various conditions. The Wilcoxon rank sum test was used to compare pairs of conditions (e.g., S0M0 versus S0M80), and the Kruskal-Wallis test was used as a non-parametric analysis of variance. The Bonferroni correction was used for multiple comparisons.

In order to quantify the contributions of the individual cues that influence ASA, e.g. onset, spectral and spatial cues, we performed multiple linear regression (MLR) analyses with threshold re T_Q_ as the output. For that analysis we tested only 0 and 80° masker locations, which were the only locations that were tested for all (eR, eC, eR, and iC) masker types. We obtained partial correlation coefficients for the three regressors: temporal onset (synchronous or asynchronous), masker type (R or C) referred to as spectral uncertainty; and spatial separation between signal and masker (0 or 80°). Each regressor was z-transformed prior to the MLR analysis. The MLR analyses were performed independently for the various signal frequencies, all-pass and band-pass maskers, and for energetic and informational maskers. Residuals for all conditions and subjects were normally distributed and had zero mean (two-tailed t-test, p>0.06). Across all subjects and conditions tested, i.e masker types, masker spectrum, and signal frequency, *R^2^*, *F*-statistic and *p*-value averaged 0.53, 43.87 and 0.002, respectively.

## Results

### Baseline All-Pass Masking Magnitudes for Co-Located Signal and Masker

**Figure 1:**
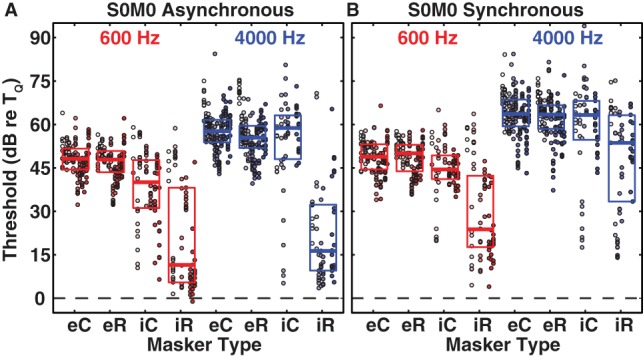
Signal detection thresholds re T_Q_, i.e. thresholds in the presence of a masker re threshold in quiet, for the all-pass maskers with the 600-Hz (red) and the 4000-Hz (blue) signals in the asynchronous (A) and synchronous (B) timing conditions for all masker types in the S0M0 (signal and masker co-located straight ahead) spatial configuration. Thresholds close to zero indicate little masking and large threshold values indicate strong masking effects. Data from individual subjects are shown as markers in different shades. The box indicates the 25^th^, 75^th^ and 50^th^ percentiles. Note that not all subjects were tested on all masker-signal combinations. To facilitate identification of individual subjects we ordered the subjects consistently throughout the plots. Subject order is: S31, S32, S33, S36, S37, S39, S42, S51, S55, S56, S57, S58, S59. eC  =  energetic constant frequency masker; eR  =  energetic random frequency masker; iC  =  informational constant frequency masker; iR  =  informational random frequency masker.

We begin by assessing the magnitude of masking by maskers co-located with the signal at 0°. In Fig. 1, as in subsequent figures, symbols indicate the repeated thresholds from each listener, with individual listeners distinguished by shades of color. Boxes indicate 25^th^, 50^th^ and 75^th^ percentiles. Masked thresholds are expressed relative to threshold in quiet, so 0 dB would indicate no masking. Red and blue indicate 600-Hz and 4000-Hz signal frequencies, respectively. Results from asynchronous and synchronous conditions are shown in Figs. 1A and B, respectively. All masker conditions showed significant masking, i.e., distributions above 0 dB (*p*<10^−6^, Wilcoxon test, for each masker condition). Masking by the energetic maskers consistently was stronger (i.e., higher masked thresholds) for 4000-Hz than for 600-Hz signals (*p*<10^−6^ Kruskal-Wallis test with correction for multiple comparisons across both timing conditions and masker types). The same was true for informational maskers (*p*<10^−6^ Kruskal-Wallis test with correction for multiple comparisons across timing condition and masker type) with the exception of the asynchronous iR masker (*p* = 0.28 Kruskal-Wallis test). Thresholds with this masker for both signal frequencies were close to threshold in quiet indicating a floor effect (see below).

In the energetic masking conditions, the various masker components were selected to fall in the same auditory filter as the signal. As expected, the two energetic masker types (eR and eC) yielded high thresholds for both signal frequencies and in both timing conditions. Threshold values for the 600-Hz and 4000-Hz signal ranged from about 32 to 64 dB and from about 40 to 84 dB, respectively. There were sizable inter-subject differences in thresholds for any particular masker condition, but within-subject variation was relatively small, typically with an inter-quartile range for each listener of ∼5 dB. Burst-by-burst variation in masker frequency composition had no significant effect on thresholds for energetic masking (*p*>0.05 for eR versus eC for 600-Hz and 4000-Hz signal frequencies and synchronous and asynchronous temporal conditions, Wilcoxon test).

In the informational masking conditions, masker components were selected to fall outside of the auditory filter containing the signal, thus reducing the contribution of peripheral masking. Any masking that occurred, therefore, would reflect processes primarily occurring within central pathways. Despite the lack of spectral overlap of signal and informational masker, masking could be as strong as in energetic-masking conditions, as was observed for the 4000-Hz constant-masker-frequency condition (no significant difference between iC and eC, *p* = 0.59 and *p* = 0.12 for asynchronous and synchronous conditions, respectively, Wilcoxon test). In all other conditions shown in Fig. 1, informational masking was significantly weaker than energetic masking (*p*<0.01 for all conditions, Wilcoxon test), but thresholds all were significantly higher than in quiet, as stated above. Informational masking also differed from energetic masking in that there tended to be greater inter-subject variability in informational than in energetic conditions. That is shown by the spread of data in Fig. 1. The distribution of data for each energetic condition tended to be unimodal, with inter-quartile ranges averaging 7 dB, whereas distributions of data for informational conditions were more widespread, with inter-quartile ranges averaging 21 dB. In a subset of the listeners informational maskers produced only minimal elevations of thresholds; specifically, in the asynchronous iR condition.

Masking tended to be stronger for synchronous than for asynchronous onsets although only the asynchronous iR and both asynchronous energetic maskers differed significantly from their synchronous counterparts (iR: *p*<0.0002, eR: *p* = 0.017 Kruskal-Wallis test with correction for multiple comparisons across frequencies). This agrees with the notion that common onsets tend to promote perceptual fusion of signal with masker. The influence of temporal cues on masking tended to be greater for informational-masking conditions than for energetic conditions. That is, across iR and iC conditions for 600-Hz and 4000-Hz signals, informational masking averaged 15 dB higher for synchronous than for asynchronous conditions, whereas the difference between synchronous and asynchronous conditions was only ∼1.5 dB and ∼7 dB for energetic masking with the 600-Hz and 4000-Hz signal, respectively.

### Spatial Release from Energetic Masking

**Figure 2:**
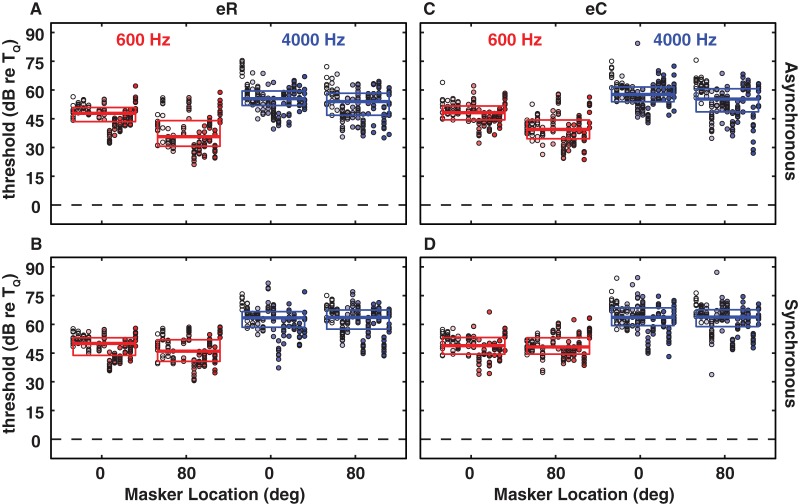
Signal detection thresholds re T_Q_ in the presence of random frequency (eR: A, B) and constant frequency (eC: C, D) energetic maskers as a function of masker location for the the 600-Hz (red) and the 4000-Hz (blue) signals in the asynchronous (A, C) and synchronous (B, D) timing conditions. All other conventions as in Fig. 1.

Decreases in masked threshold associated with spatial separation of signal and masker are referred to as spatial release from masking. We begin with the results from the energetic maskers. Fig. 2 shows data for eR (asynchronous: A; synchronous: B) and eC (asynchronous: C; synchronous: D) maskers at 0 and 80° masker locations. Data for S0M0, i.e. signal and masker co-located straight ahead, are re-plotted from Fig. 1. The general characteristics of energetic masking described for the S0M0 condition in Fig. 1 also were observed in the S0M80 condition.

Our free-field energetic masking conditions were somewhat analogous to dichotic (i.e., head-phone) stimulation conditions used in the large body of research on binaural masking-level differences (BMLDs). In the so-called S0Nπ condition in which a diotic sinusoidal signal (i.e., equal at the two ears) is masked by a dichotic broadband noise with a 180° phase difference between the ears, one typically finds BMLDs to be ∼10–15 dB for a 600-Hz signal [19]. However, one needs to note that in those studies the signal is perceived at the center of the head and the masker elicits a diffuse rather than localized percept as is the case with our free-field stimuli. Additionally, the comparison can only be applied to low-pass signals (<∼1.5 kHz), for which phase cues are preserved. For high-frequencies, like our 4000-Hz signal, phase information cannot be accurately encoded by the auditory system and instead ILDs are the main cue in the free-field. Instead of using the term BMLDs we refer to spatial release from masking here since we employed free-field stimuli. We compute spatial release from masking by subtracting the median threshold of the S0M80 (or any other spatial) configuration from the thresholds of the S0M0 configuration. Despite the differences between free-field, multi-tone maskers and closed-field, broadband noise maskers, at least the results for the asynchronous timing condition seem to be in agreement with the values reported for BMLDs with a 600-Hz signal. We found a statistically significant spatial release for the 600-Hz signal equal to 12 dB for the asynchronous eR masker (*p*<10^−6^, Wilcoxon test) and equal to 9 dB for the asynchronous eC masker (*p*<10^−6^, Wilcoxon test). For all other conditions spatial release never exceeded 2 dB and S0M0 vs S0M80 threshold distributions were not statistically different from each other (*p*>0.025, Wilcoxon test). These results accord with the notions that spatial release from energetic masking is quite weak, that it can be outweighed by temporal cues (i.e., common onsets) and that spatial release from energetic masking is greater for low-frequency than for high-frequency signals.

### Spatial Release from Informational Masking

**Figure 3:**
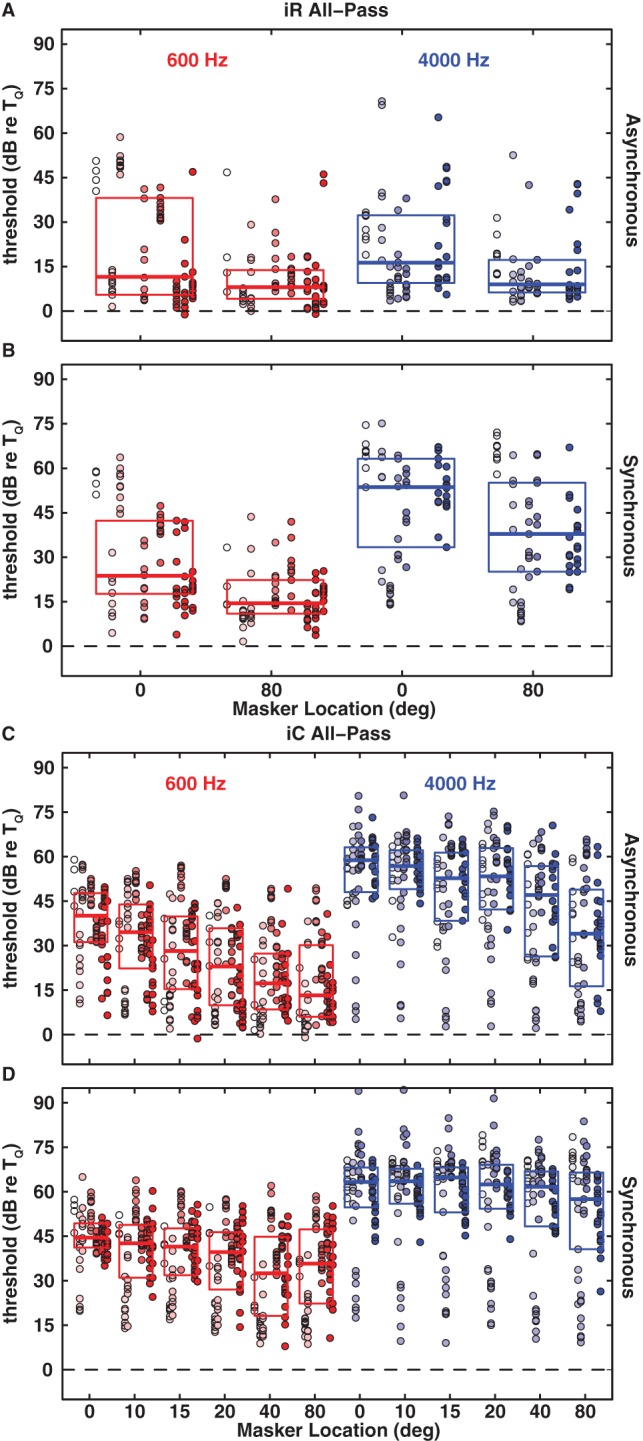
Signal detection thresholds re T_Q_ in the presence of random frequency (iR: A, B) and constant frequency (iC: C, D) all-pass informational maskers as a function of masker location for the the 600-Hz (red) and the 4000-Hz (blue) signals in the asynchronous (A, C) and synchronous (B, D) timing conditions. All other conventions as in Fig. 1. Note that we tested more spatial configurations for the iC masker.

Spatial release from informational masking in most cases was greater than for energetic masking. Results obtained with broadband maskers are shown in Fig. 3; conventions are the same as in Fig. 2, although more spatial configurations were tested for the iC masker. Again the masked thresholds for the 4000-Hz signal consistently were higher than the corresponding thresholds for the 600-Hz signal (*p*<10^−6^, Kruskal-Wallis test with correction for multiple comparisons per type and timing condition, except asynchronous iR masker: *p* = 0.06, floor effect). Thresholds for individual listeners were fairly consistent across repeated measurements, with inter-quartile ranges averaging 9 dB across all the listeners and all the conditions shown in Fig. 3. Inter-listener variability, however, was in most conditions larger than for energetic masking. In the iR asynchronous S0M0 600-Hz condition (Fig. 3A), for instance, three of the listeners experienced around 45 dB of informational masking, whereas the other five listeners experienced less than 15 dB of masking. In some conditions, the inter-listener variability, as indicated by the inter-quartile ranges, was relatively small. We attribute that small variability to ceiling (ceiling (e.g., iC, S0M0) and floor (e.g., asynchronous iR, S0M80) effects.

Introduction of a 80° spatial separation between the signal and the random-informational masker (iR) resulted in significant spatial release from masking of the 600-Hz signal. Spatial release was 4 dB (*p*<0.003, Wilkoxon test) for the asynchronous condition and 9 dB (*p*<10^−6^, Wilkoxon text) for the synchronous condition. For the 4000-Hz signal, masking release was larger than for the 600-Hz signal although *p*-values were higher (7 dB, *p*<0.001 for asynchronous; 16 dB, *p* = 0.01 for synchronous, Wilkoxon test). In the constant-masker-frequency (iC) condition the 600-Hz signal yielded larger amounts of spatial masking release than the 4000-Hz signal. The asynchronous timing condition with the 600-Hz signal exhibited a systematic decrease in threshold (i.e., increase in masking release) with increasing signal/masker separation. Significant spatial release from masking was observed in that condition for signal/masker separations as small as 15° (12 dB, *p* = 0.0002; comparison of S0M15 versus S0M0, Kruskal-Wallis test with correction for multiple comparisons). In contrast, for the 4000-Hz signal in the iC condition, only separations greater than 40° configuration were significantly different from S0M0 in the asynchronous condition (12 dB, *p*<0.0001 for S0M40 versus S0M0 and 25 dB, *p*<10^−6^ for S0M80 versus S0M0, Kruskal-Wallis test after correction for multiple comparisons). Spatial release from informational masking was somewhat weaker in the synchronous than in the asynchronous timing condition. There was no spatial release from masking of the 4000-Hz signal for any signal/masker separation in the synchronous condition (*p* = 0.12, Kruskal-Wallis test after correction for multiple comparisons). In the synchronous condition with the 600-Hz signal we found significant spatial release for a spatial separation of 20° (*p*<10^−5^, Kruskal-Wallis test across all masker locations; 5 dB, *p* = 0.003 for S0M20 versus S0M0 after correction for multiple comparisons).

For the 600-Hz signal, across all the signal/masker separations in the iC condition, spatial release was significantly less in the synchronous condition than in the corresponding asynchronous condition (*p* = 0.003, Kruskal-Wallis test with correction for multiple comparisons). Note that in the synchronous condition, signal and masker components presumably were fused perceptually by common onsets, whereas that was less the case in the asynchronous condition. Despite that perceptual fusion, however, an 80° separation of signal and masker produced ∼9 dB of spatial release even in the difficult constant-masker-frequency, synchronous-onset condition, indicating that spatial cues for segregation could overcome spectral and temporal cues for integration. In the iR condition, spatial release was actually greater in the synchronous than in the asynchronous condition. We attribute that to a floor effect in the asynchronous iR condition in that there was relatively little informational masking in the S0M0 configuration, so there was little room for improvement in detection in the S0M80 configuration.

Significant spatial release from masking of the 600-Hz signal was observed for every tested signal/masker separation > = 20°. In contrast, significant release of informational masking of the 4000-Hz signal was seen only for the iC asynchronous 80° separation condition. The influence of spectral region on spatial release from masking is explored further in the next section.

### Spatial Masking Release from Masking in Low- versus High-Frequency Bands

**Figure 4:**
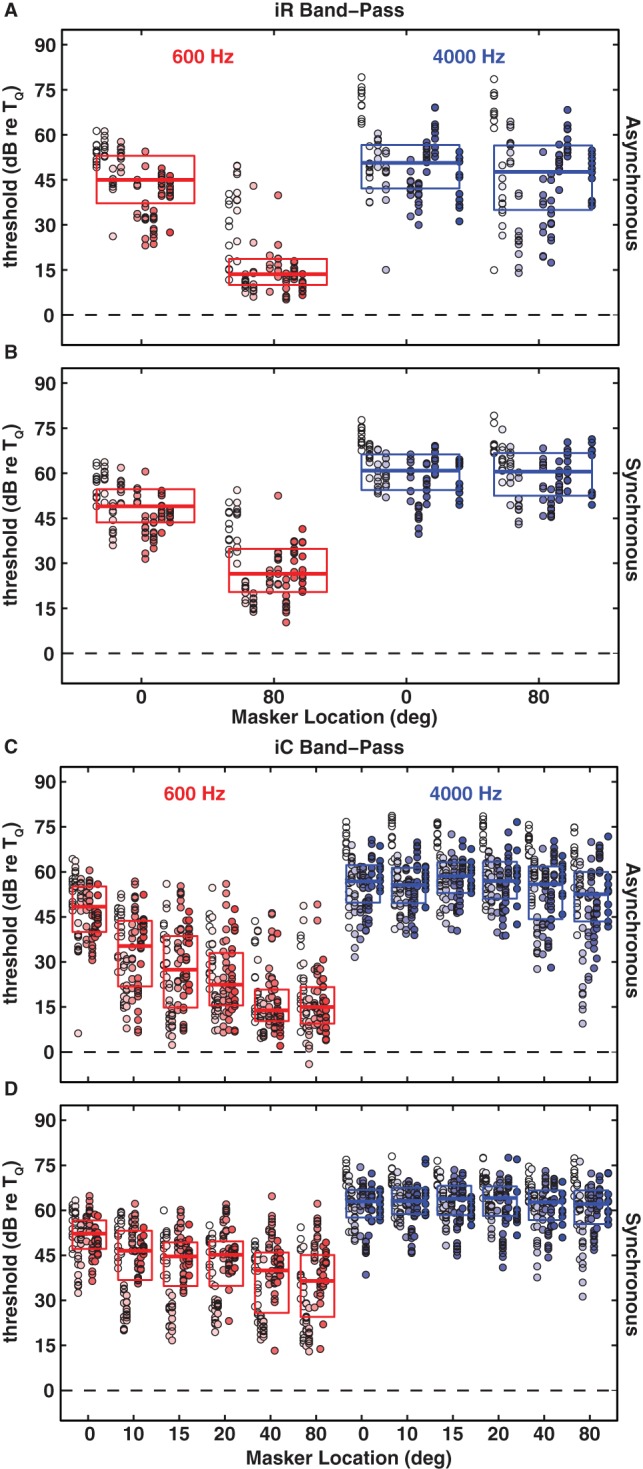
Signal detection thresholds re T_Q_ in the presence of random frequency (iR: A, B) and constant frequency (iC: C, D) band-pass informational maskers as a function of masker location for the the 600-Hz (red) and the 4000-Hz (blue) signals in the asynchronous (A, C) and synchronous (B, D) timing conditions. All other conventions as in Fig. 1. Note that we tested more spatial configurations for the iC masker.

In the All-Pass-Masker condition the masker covered both the low-frequency range (<∼1500 Hz) containing the 600-Hz signal, in which ITDs are the dominant spatial cue, and the high-frequency range (>∼3000 Hz) containing the 4000-Hz signal, in which ILDs are the dominant cue. For the purpose of isolating ITD and ILD contributions to spatial release from masking, we constructed low-pass maskers with components limited to 200–1500 Hz and high-pass maskers with components limited to 1.5 to 12 kHz. Informal pilot tests of the 600-Hz signal with the high-pass masker and the 4000-Hz signal with the low-pass masker demonstrated essentially no masking. For that reason, we conducted the formal tests of 600- and 4000-Hz signals only with low- and high-pass maskers, respectively. In each case, masker components were excluded from the band spanning 1/3 octave above and below the signal frequency. Figure 4 presents the results in the same format as in previous figures.

For nearly every masker type, asynchronous versus synchronous, and every signal/masker separation, the band-pass maskers produced substantially more masking than did the all-pass maskers (*p*<0.004, Kruskal-Wallis test with correction for multiple comparisons). In particular, the band-pass iR maskers across all conditions, yielded masked thresholds averaging ∼22 dB higher than the corresponding all-pass iR maskers (Fig. 4). Remarkably, the magnitude of masking by both band-pass iR maskers was as great as that by the corresponding energetic maskers (p>0.05, Kruskal-Wallis test) (Fig. 2). Also, the inter-subject variability was substantially less in the band-pass conditions (mean inter-quartile ranges ∼12 dB) than in the all-pass conditions (mean inter-quartile ranges ∼23 dB). Similar to the results with the all-pass maskers, both low- and high-pass conditions showed overall higher thresholds in the synchronous timing conditions than in the corresponding asynchronous thresholds (*p*<10^−5^, Kruskal-Wallis test corrected for multiple comparisons). Also, greater masking was observed for all high-pass conditions compared to all low-pass conditions (*p*<10^−5^, Kruskal-Wallis test corrected for multiple comparisons).

The results for spatial release from masking by the low- and high-pass maskers replicated and, in some cases, amplified the results obtained for the respective low- and high-frequency signals masked by the all-pass maskers. Spatial release from informational masking was largely limited to low frequencies. Masking of the 600-Hz signal with the low-pass masker showed significant spatial release in both timing conditions, both (iR and iC) masker types, and all spatial configurations (*p*<10^−5^, Kruskal-Wallis test with correction for multiple comparisons). For both timing conditions with the iC masker significant spatial release was observed for a 10° signal/masker separation, which was the smallest separation tested (∼13 dB, *p*<10^−6^ asynchronous and ∼6 dB, *p*<0.0001 synchronous, Kruskal-Wallis test with correction for multiple comparisons). At a spatial separation of 80° spatial release was as large as 23 dB and 16 dB for synchronous iR and iC maskers, respectively. In the asynchronous timing condition these values increased to 33 dB and 34 dB for the iR and iC maskers, respectively. For the 4000-Hz signal, in contrast, there was no significant spatial release from the high-pass masker for either (iR or iC) masker type, either asynchronous versus synchronous timing conditions (*p*>0.3, Kruskal-Wallis test with correction for multiple comparisons), or spatial configurations. Note, however, that some individual listeners were able to profit from large spatial separations (>40°) even with the 4000-Hz signal and high-pass maskers.

### Summary of Spatial Release from Masking

**Figure 5:**
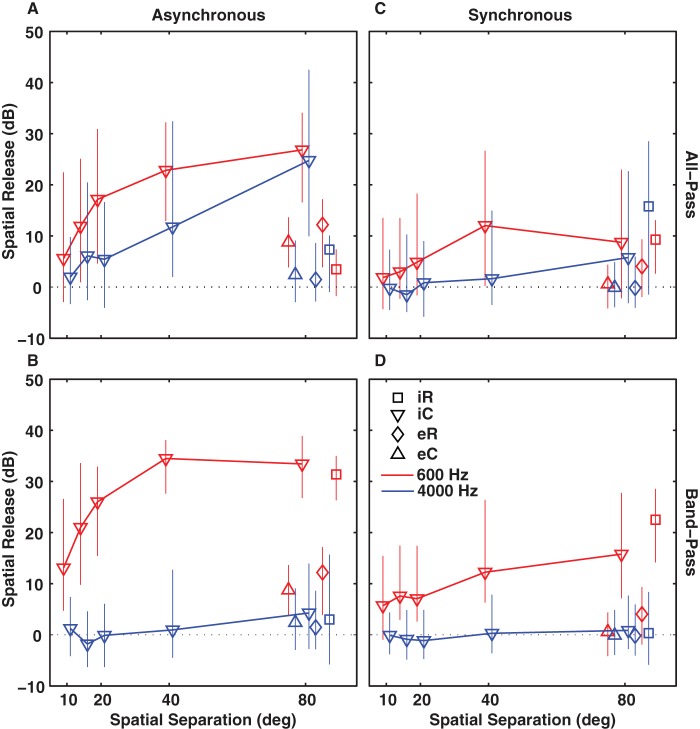
Spatial release from masking as a function of spatial separation between signal and masker. We obtained spatial release by subtracting detection thresholds for all spatial configurations from the S0M0 thresholds. Spatial release for the 600-Hz signal and the 4000-Hz signal are plotted in red and blue, respectively. Squares and diamonds represent iR and eR masker data, downwards and upwards pointing triangles iC and eC maskers, respectively. Data for the all-pass masking backgrounds are shown in panels A and C, band-pass data in panels B and D. Results for the asynchronous and synchronous timing conditions are plotted in panels A, B and C, D, respectively. Error bars indicate 25^th^ and 75^th^ percentiles. Note that for a given timing condition the same energetic backgrounds are replotted in the corresponding all-pass and band-pass panels. Frequencies for the energetic backgrounds per definition always fall within the protected-band rendering a distinction in all-pass and band-pass impossible.

In Fig. 5 we summarize the spatial release from masking that was observed across various conditions. Asynchronous, synchronous, all-pass, and band-pass conditions are shown in the various panels, low and high frequency ranges are indicated by colors, and all other energetic and informational masking conditions are indicated with various symbols. Each plotted value is the median spatial release for S0Mx and error bars indicate IQRs. Note that for a given timing condition the same energetic backgrounds are re-plotted in the all-pass and band-pass panels.

If we first focus on data obtained in the asynchronous timing condition for the all-pass and band-pass maskers (Fig. 5AB) we see that as before the all-pass iR masker (squares) resulted in little spatial release for either signal frequency since masked thresholds in the S0M0 configuration were already close to T_Q_ (floor effect). In contrast, the band-pass condition with the 600-Hz signal (red) showed a spatial release of ∼31 dB, similar to the value found with the iC masker.

Spatial release for both all-pass and band-pass iC maskers with the 600-Hz signal (red) followed a function that began to saturate at 40°, whereas spatial release for the 4000-Hz signal (blue) seemed to increase linearly with increasing signal and masker separation. There was no significant spatial release with the band-pass iC masker for the 4000-Hz signal (Fig. 5B). It is also noteworthy that with energetic maskers (upwards pointing triangles) spatial release never exceeded 15 dB.

The synchronous maskers (Fig. 5CD) mirrored the trends in the asynchronous data. Overall spatial release was much smaller with the 600-Hz signal (<20 dB) and absent with the 4000-Hz signal when compared to the corresponding asynchronous conditions but the saturating trend for the iC still was evident. Large spatial separations (≥40°) could result in spatial unmasking of low-frequency signals in synchronous maskers that was at least as large as the values described for energetic conditions in the BMLD literature [19] or obtained with asynchronous energetic masking backgrounds in the present study.

### Quantifying the Weighting of Temporal, Spectral and Spatial Cues

We performed a multiple linear regression analysis in order to obtain partial correlation coefficients (weights) for temporal onset, masker type (spectral uncertainty) and spatial separation between signal and masker (spatial cues).

**Figure 6:**
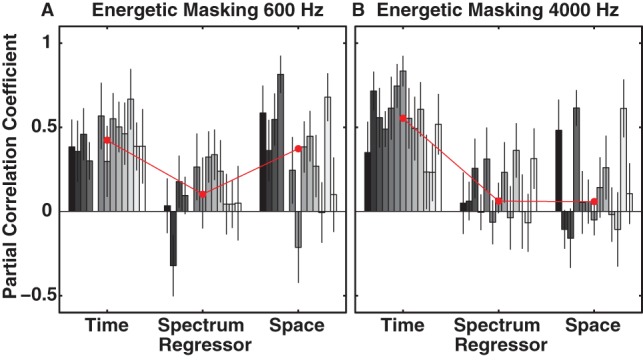
Partial correlation coefficients (weights) for regressors temporal onset (time), masker type, i.e. eC and eR (spectrum), and spatial separation between signal and masker (space) as obtained by multiple linear regression. Results for the 600-Hz and 4000-Hz signal with energetic maskers for individual subjects (bars in different shades of gray) are shown in A and B, respectively. Error bars indicate 95% CIs and the red line with circular markers depicts the median across all subjects. Values close to 1 (0) indicate a strong positive (weak) weight of a given regressor. Subject order as in 1.

**Figure 7:**
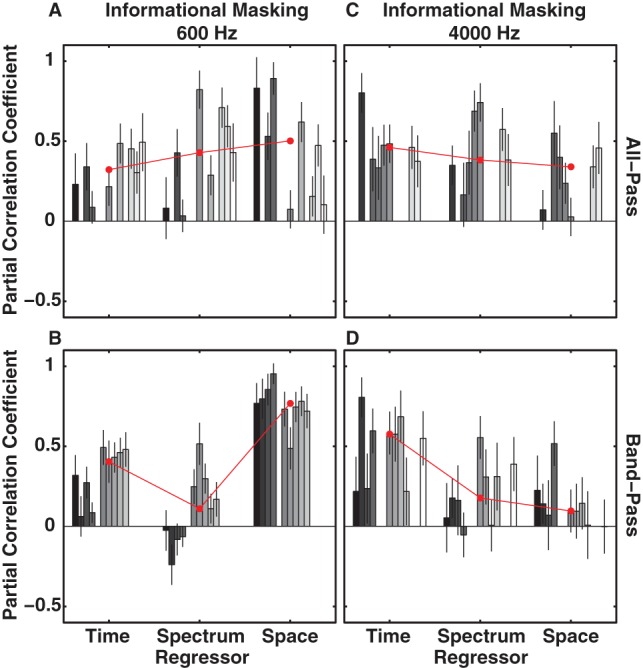
Partial correlation coefficients (weights) for regressors temporal onset (time), masker type, i.e. iC and iR (spectrum), and spatial separation between signal and masker (space) as obtained by multiple linear regression. Results for the 600-Hz (A, B) and 4000-Hz (C, D) signal with informational all-pass (A, C) and band-pass (B, D) maskers for individual subjects (bars in different shades of gray) are shown. Error bars indicate 95% CIs and the red line with circular markers depicts the median across all subjects. Values close to 1 (0) indicate a strong positive (weak) weight of a given regressor. Subject order as in 1.

Fig. 6 shows the results of the multiple linear regression analysis for energetic maskers with the 600-Hz (A) and 4000-Hz (B) signals. The bars in different shades of gray indicate values for individual subjects ordered in the same way as in previous figures to facilitate comparison. Error bars indicate 95% CIs and the red line with circular markers depicts the median across all subjects. In the case of the 600-Hz signal (Fig. 6A) temporal and spatial cues yielded roughly equal weights close to 0.5 across most of the subjects, whereas the weights for the spectral uncertainty were close to 0. In two subjects (S31, S36) spatial weights were even larger than the corresponding temporal weights. Subject 42 was a notable exception in that respect. In agreement with the absence of any spatial release from energetic masking (see also Fig. 2) her spatial weights were negative albeit not significantly different from 0 (95% CIs overlapped with 0). This indicates that she was not using spatial cues to solve the task. The weights for spectral uncertainty seemed to vary across subjects while 7 out of 12 subjects did not seem to use those cues (CIs crossing 0); the other subjects exhibited values that could be as large as their corresponding spatial weights (S39) but that never exceeded them. It is interesting to see that on a population basis threshold distributions for the eR and eC maskers were not significantly different from each other (Fig. 2) indicating the equivalence of the two masker types. However, based on the weights shown in Fig. 7A spectral uncertainty seems to account for part of the data of some subjects. Taken together these results seem to support the notion that ITDs are effective in creating spatial release from energetic masking as for example discussed in the BMLD literature [19]. Also, individual subjects may employ different listening strategies and may weigh cues present in the stimuli differently.

The results for the 4000-Hz signal data (Fig. 6B) clearly differ from the 600-Hz-signal results in that temporal cues outweigh spectral uncertainty and spatial cues if considered across all subjects (median indicated by the red line). Averaged across listeners, energetic masking is dominated by onset synchrony and shows negligible influence of spectral uncertainty or space. Weights for individual subjects, however, could vary. For example in S31 and S36 values for the spatial cues were at least as large as the corresponding temporal cues. Moreover, similar to the 600-Hz data, spectral uncertainty cues were equal to 0 in 6 of the 10 subjects tested and differed from 0 in the remaining 4 subjects. Spectral uncertainty weights could even be larger than the corresponding spatial cues (S33, S37, S56). Overall, when compared to the 600-Hz data and in agreement with the absence of any spatial release for the 4000-Hz signal with energetic maskers (Fig. 5 upwards pointing triangles and diamonds), weights for the temporal cues were on average much larger than the weights for spectral uncertainty and spatial cues.

Next, in Fig. 7, we describe the weights obtained with informational masking backgrounds. Weights for the 600-Hz signal and 4000-Hz signal with all-pass backgrounds are shown in panels A and C, respectively. When comparing the two all-pass conditions across all listeners it becomes apparent that the weights generally follow inverse trends for the 600-Hz signal and the 4000-Hz signal. Whereas the values of the weights for temporal, spectral and spatial cues gradually increase (in that order) for the 600-Hz data, values for the 4000-Hz data decrease, with weights around 0.5 and 0.3 for temporal and spatial cues, respectively. As seen before with energetic maskers (Fig. 6) individual subjects could deviate from the trend observed across the population (e.g. S42 and 59). Interestingly, weights for spectral uncertainty cues were on average quite high for both signals. In agreement with the finding that iR backgrounds yielded less masking than iC backgrounds (Fig. 5) this indicated that subjects were able to profit from the spectral difference between the two types of IM maskers in the all-pass condition. The weights for the spectral uncertainty cues for the band-pass maskers with the 600-Hz signal (Fig. 7B) and the 4000-Hz signal (Fig. 7D) on the other hand were on average smaller than their all-pass counterparts.

Across all conditions tested (including the energetic maskers) we observed the largest median weights for spatial weights with the 600-Hz signal and low-pass backgrounds (0.8). In this condition spatial cues, i.e. predominantly ITDs, even outweighed temporal cues that are thought to be the strongest grouping cues [1], [20]. On the other hand temporal weights were largest (even when compared to the other IM maskers) for the 4000 Hz signal with the high-pass background. This held true even when comparing between individual subjects where possible. Weights for spectral uncertainty and spatial cues were both below 0.2 contributing only marginally to the explanation of the observed data. This seems to suggest that ILDs, which dominate in the high-pass condition, were deemed less salient by the auditory system of our subjects than temporal onset cues.

## Discussion

The results support the notion that spatial separation of a non-speech signal and a non-speech masking background can strongly facilitate signal detection. In our paradigm, signal detection should be enhanced if signal and background formed separate auditory objects, i.e. they were perceptually segregated (fission). We demonstrated that if both signal and background fell within the low-frequency range (<1.5 kHz) the benefit from spatial separation was largest for all conditions tested in our study even if spectro-temporal cues favored perceptual fusion. Subjects informally reported that even at low signal levels and small spatial separations they were able to hear two distinct and localized auditory objects, i.e. the signal straight ahead and the background at a different location. Although it is highly unlikely that in natural environments two distinct sources at disparate locations will start synchronously, as was the case with our synchronous maskers, our data show that the auditory system is capable of weighting spatial cues more strongly than onset cues, which have been deemed to be among the strongest cues for grouping [1], [20]. In informational-like conditions listeners potentially could benefit greatly even from small spatial separations of two sound sources if the signal frequency is relatively low (e.g. formants in speech). In the all-pass condition a separation of roughly 20° yielded significant spatial release that was as large as the spatial release obtained with energetic maskers separated by 80°. In the band-pass condition this was already the case for a separation of about 15°. We conclude that for the stimuli used here, fine structure ITDs are stronger cues for ASA than are ILDs or the monaural head-shadow effect, even in the presence of competing spectro-temporal cues.

Surprisingly, compared to the all-pass condition, thresholds with either band-pass iR masker in the S0M0 configuration were high. As with the iC masker only with the 600-Hz signal did subjects benefit from a 80° spatial separation. It is not clear how to interpret this finding based on knowledge of spatial cues or principles discussed in the informational masking literature [15]. For example, listeners did not seem to benefit from the monaural head-shadow effect for the 4000-Hz signal. Based on measurements of head-related transfer functions [21] and with a separation of 80° between signal and masker one would expect a monaural benefit of ∼10 dB and ∼15 dB for frequencies between 4–8 kHz and 10–14 kHz, respectively. This amount of release is clearly not seen with the high-pass maskers and 4000-Hz signal. There is considerable agreement, at least in the speech perception literature, that the better-ear advantage can often explain a large proportion of the observed masking effect [2]. The current data are an interesting exception to that rule [12] and are indicative of other than spatial processes contributing to the observed detection thresholds.

In addition, the band-pass data are likely related to the question of the effective range for spectral integration [22]. At more peripheral levels of the auditory system, spectral integration can be modeled based on cochlear filter properties. How far these models are also applicable to central processes is currently unclear. But stimuli like those used in the present study seem to be suited to address this question.

Spectral integration is also important with regard to the use of tones rather than noise as energetic maskers. It could be argued that the masking seen with these tonal energetic maskers might in part be due to informational masking. Although most likely present, the contribution of informational masking will be small since 1) the amount of masking with our tonal energetic maskers is comparable to the amount reported for noise maskers, 2) spatial release from masking is negligible with our energetic maskers as is the case for noise maskers but, as we show, not for informational maskers in comparable situations, and 3) we did not find any threshold differences between the two types of energetic maskers, i.e. eR and eC, although the two maskers are perceptually distinct. Accordingly, we would like to conclude that informational masking contributed little or nothing to the masking seen in our energetic conditions.

Next, we consider the possibility that listeners could have detected the signal based on loudness cues alone, instead of perceptually segregating signal from masker. These cues would have been most salient for the energetic maskers. Indeed informal listening confirmed that listeners did not perceive two distinct auditory objects in energetic masking conditions. Signal and masker were fused but the interval containing the signal sounded louder than the masker-only interval for supra-threshold levels. For these stimuli fusion of signal and masker was mandatory forcing listeners to resort to the use of loudness cues. For the informational stimuli, on the other hand, it is hard to see how loudness cues could have aided the listeners in achieving thresholds that in some instances approach those seen for signal in quiet. Similarly, it is difficult to see how loudness cues could account for the prominent differences between energetic and informational conditions with the co-located R and C maskers in regard to thresholds and inter-subject variability. That is, thresholds with the iR maskers were considerably lower than those for iC and for energetic maskers and even approached signal-in-quiet values. Moreover, inter-subject variability with both the iR and the iC maskers was greater than the variability seen with energetic maskers, with some subjects exhibiting thresholds well below those seen for the energetic maskers. Our own listening experience with informational maskers and informal discussions with our subjects indicate that in these conditions two distinct auditory objects were perceived until threshold is reached. For these reasons, and to avoid interference with the tracking algorithm, we chose not to rove the masker levels.

Although to our knowledge this study is the first to test independently the influence of signal and background frequency composition and spatial cues on signal detection thresholds in a free-field, several previous studies are directly related to and inspired our study. For example Kidd and colleagues [17] introduced the original version of the stimuli used here and studied the effect of changing ITD [17] or spatial separation [12] of signal and masker under head-phone or free-field stimulation, respectively. Under headphone stimulation and informational masking conditions with a 1000-Hz signal and a synchronous 4-component background that ranged in frequency from 200 to 5000 Hz, Kidd and colleagues [17] found on average an advantage of about 18 dB and 13 dB when presenting signal and masker dichotically compared to monotically for iC (their MBS) and iR (their MBD) maskers, respectively. Comparable to our study, inter-subject variability was large, e.g. one subject showed an advantage as large as 35 dB with iC maskers and very low thresholds for iR maskers and did not profit from manipulations of the inter-cranial sound image. Due to the use of headphone stimulation it is difficult to compare these results directly with our free-field data.

A second study by Kidd and colleagues [12] using free-field stimuli, however, seems to be more amenable to comparison. That studied used a pattern detection task with informational maskers and various synchronous signal patterns (e.g. ascending or descending in frequency) spanning frequency ranges from 200 Hz to about 6500 Hz. Interestingly, and in contrast to our results, a large improvement of 35 dB (180° spatial separation, 2 of 3 subjects) occurred only in the frequency range from 2910 to 6540 Hz. For the lower frequencies spatial release was about 20 dB across all subjects. Kidd and colleagues [12] concluded based on measurements of the head-related transfer functions in their three listeners that the head-shadow effect did not play a role in the large improvements seen in their data for informational maskers. Again it is difficult to directly compare our data to theirs due to differences in task (2 interval 2-AFC vs 1 interval 6-AFC), stimulus composition (pulsed constant frequency signal vs frequency patterns), masker frequency range (low-pass, high-pass and all-pass vs constant 200 to 6500 Hz range) and spatial configurations (only right hemifield up to 80° vs across hemifields ranging from −90° to 90°). We would nevertheless like to offer the following comments. Whereas the low-frequency informational masking data are in rough agreement between the two studies, the high-frequency data are not. Across our population we did not find large amounts of spatial release from masking neither with high-pass nor with the all-pass backgrounds. However, some of our subjects showed spatial release that is of the same order of magnitude as the one reported by Kidd and colleagues [12] for similar spatial separations. It is also interesting to observe that with low-frequency signals all three subjects in the study by the Kidd group performed very well while with the high-frequency signal only two subjects showed large spatial release and their third subject's thresholds improved only by roughly 10 dB for separations >90°. This might indicate in agreement with our own results that the low-frequency signal condition was overall easier and that in the high-frequency signal condition subjects exploited the available cues differently.

In addition to the differences for the informational masking data, results for energetic masking also differ between the Kidd group [12] and our data. The former found spatial release of 10 dB with high frequency signals using a broadband noise masker for spatial separations >120°, whereas we reported the same amount of spatial release for our low-pass signal at 80° separation and energetic maskers but no significant release for high-pass signal and maskers. It is not clear how to reconcile these findings. Whereas our data agree for example with head-phone BMLD experiments [19], the data from the Kidd group seem to be in agreement with the amount of spatial release expected by the head-shadow effect. Here, we note that our use of tonal maskers to assess the amount of energetic masking in the context of ASA is unlike the more conventional use of noise maskers. Our rationale was to create energetic maskers that were as comparable to the informational masker with respect to their temporal and, to some degree, to their frequency composition as possible. However, we deem it unlikely that this difference in masker composition (tone complex vs broadband noise) can fully account for the observed differences in the two studies.

Notwithstanding these differences, both studies seem to indicate that the mechanisms for spatial release from energetic and informational masking are due to different mechanisms that call for different models of the two processes [13]. Within-channel binaural analysis mechanisms, like for example the equalization-cancellation model [23], that work well for energetic maskers fail to account for thresholds observed in ASA paradigms. Models that are either based on the stimulus statistics [24] or that are inspired by neuronal processes [25] are more likely to succeed in predicting spatial release found in ASA paradigms.

Several authors report that binaural processing plays only a minor role in ASA [9], [10]. Studies using speech stimuli [26], rhythmic release from masking paradigms [27], [28] and synthetic sounds with naturalistic spectro-temporal properties [11] suggest that spatial cues, especially ITDs, are not important for ASA and are outweighed by temporal cues. The present results as well as another recent study from our laboratory [14] are in disagreement with that conclusion. We can think of several reasons for this discrepancy in results. In general, the presence of energetic masking is difficult to rule out with natural speech, even with careful stimulus design. Although speech is likely the most important stimulus in daily life it is somewhat difficult to control and parameterize in a laboratory setting. On the other hand one might argue that the artificial stimuli used here are not representative of the real world situation that they are supposed to model and are therefore irrelevant. However, it should be considered that analytical stimuli might help to dissect individual computations of the process that is ASA. Here we were particularly interested in the relative weights the auditory system puts on the ASA cues present in our artificial stimuli. This would not have been readily possible with speech stimuli. However, as Turgeon and colleagues [27] concluded, additional experiments with more naturalistic stimuli need to be performed “to determine to what extent these results are generalizable to the perceptual organization of complex sounds”.

At least one attempt at using such stimuli has been reported in the literature [11]. Under head-phone stimulation and making use of parametrically well defined stimuli that mimic the spectro-temporal statistics of natural sounds, Schwartz and colleagues demonstrated that within their framework ITDs do not significantly contribute to the identification of sound elements in a mixture but enable listeners to localize individual elements. We neither tested directly the identifiability nor the localizability of the signal. We note, however, that our results with the 600-Hz signal seem to disagree with the finding of Schwartz and colleagues. It could be argued that in the presence of informational maskers signal detection is akin to signal identification since for a successful detection the auditory system has to segregate signal from masker and thus identify the signal. Our finding of large amounts of spatial release from masking with the 600-Hz signal would therefore argue for the importance of ITDs as segregation and identification cues. We think that apart from the difference in stimulus presentation (free-field vs head-phones) and metrics, task and stimulus specifics hold the key to a possible explanation of this discrepancy as is also acknowledged by Schwartz and colleagues in their Discussion with regard to previous studies [11; p. 366, second paragraph]. The signal in their study did not differ from the maskers since it was drawn from the same distribution. It only became apparent (popped out) because it was the only repeated sound element in the sequence. The task was therefore to detect a repeating but *a priori* unknown and varying sound element, rather than detecting a well-known signal, as was the case in most of the other studies cited here including our own. This, in combination with a different masker frequency range (200–1500 Hz vs 40–3967 Hz) and the artificial head-phone stimulation, might explain discrepancies between their and our results.

Lastly, to make an attempt at linking behavior to its neuronal substrates, we would like to allude to the neurophysiological finding that various fields in cat auditory cortex are sensitive to the spatial location of sounds [29]. But at the same time lesion studies have shown that not all of these areas are involved in sound localization behavior [30]. It is therefore possible that sound localization and ASA make use of spatial cues in different ways [31], [32], [14]. Does grouping operate on individual cues (parameter) or on a “space map” (feature)? It is viable that for ASA spatial cues are processed independently per frequency in contrast to the proposed across-frequency integration for sound localization [33]. Although we did not test this hypothesis directly, nevertheless, we deem it an interesting question for future psychophysical and neurophysiological research.
